# The role of minority stress in disordered eating: a systematic review of the literature

**DOI:** 10.1007/s40519-024-01671-7

**Published:** 2024-06-08

**Authors:** Fabrizio Santoniccolo, Luca Rollè

**Affiliations:** Department of Psychology, University of Turin, Via Verdi 10, 10124 Torino, TO Italy

**Keywords:** Minority stress, LGBT, Disordered eating, Eating disorders, Systematic review

## Abstract

**Purpose:**

Sexual and gender minorities (SGMs) show a heightened risk of disordered eating compared to heterosexual and cisgender people, a disparity which may be caused by exposure to minority-specific stressors, such as discrimination and violence. This systematic review aims to summarize available evidence on the role of minority stress in disordered eating and SGM-specific aspects.

**Methods:**

Following PRISMA guidelines, scientific search engines (EBSCO, PUBMED, Web of Science) were screened up to 31st of January 2024, including English-language original research papers containing analyses of the relationship between minority stress and disordered eating. 2416 records were gathered for screening. After application of inclusion and exclusion criteria, thematic analysis was conducted regarding 4 research questions: effects of minority stress on disordered eating, mediating factors, specificities of SGMs and differences between identity categories.

**Results:**

30 studies were included. Several aspects of minority stress are reliably associated with different forms of disordered eating. The relationship between minority stressors and disordered eating is mediated by aspects such as shame, body shame, or negative affect. SGMs show several specificities, such as the presence of a role of LGBTQIA + communities and additional gender-related pressures. Bisexual people and gender minorities appear to feature comparatively higher risks, and gender-related factors shape paths leading to disordered eating risk.

**Conclusion:**

Minority stress is an important predictor of disordered eating, making SGM people’s health particularly at risk. Institutional and organizational anti-discrimination policies are needed, as well as further research. Clinical interventions may benefit from exploring and incorporating how minority stressors impact SGM people.

*Evidence level* I—Systematic review.

**Supplementary Information:**

The online version contains supplementary material available at 10.1007/s40519-024-01671-7.

## Introduction

Disordered eating behaviors can severely impact short and long-term physical and mental health, significantly reducing one’s quality of life [1, 2]. When manifesting as full-blown eating disorders, the impacts for the individual and the burden these behaviors impose on familial and healthcare systems are particularly heavy even when compared to other mental illnesses, posing a relatively high risk of mortality and heightening the number of years lived with disability [3].

People that are part of Sexual and Gender Minorities (SGMs) seem to be particularly at risk—that is, people who have either a sexual orientation other than heterosexual (e.g., gay, lesbian, bisexual, pansexual^1^) or a gender identity that is not consistent with one’s assigned gender at birth (e.g., trans, non-binary, gender non-conforming). The prevalence of disordered eating has been found to be higher in SGMs when compared to heterosexual or cisgender people [6–9]. SGMs have also been found to be at significant risk for body image concerns, such as increased body dissatisfaction [10, 11], weight bias [12] and weight dissatisfaction [13, 14], which are closely linked to disordered eating.

Processes that lead the individual to develop disordered eating behaviors have been extensively studied regarding cultural and social influences, leading to the development of contributions such as the Tripartite Influence Model [15, 16] and Objectification Theory [17, 18]. Additionally, factors tied to a person’s identity have been shown to play an important role as well. The last few decades of research have highlighted how SGMs appear to be at a heightened risk of developing psychopathology compared to heterosexual and cisgender people [19]. This has been hypothesized to be due to exposure to additional stressors that are specific to SGMs, such as discrimination and violence [20–22]. Reviews of studies [23–25] report that the assumptions and hypotheses of the minority stress model have found significant empirical support for how they relate to disordered eating as well.

Exposure to these stressors has further been hypothesized to determine an elevation in general psychological processes, such as coping and emotion regulation processes, social and interpersonal processes, as well as cognitive processes [26], a psychological mediation framework that may explain how exactly these stressors translate to a heightened risk of general psychopathology. Mediation analyses of minority stress elements uncover the processes by which these stressors act, discovering the internal psychological mechanisms they are linked to.

Additionally, one’s identity as SGM often implies differences and specificities in one’s experiences [27, 28]. Despite several conflicting areas, past research suggests one’s experience with body image and disordered eating may vary depending on the specific identity of the person in regard to sexual orientation and gender identity [9, 29, 30], such as differing appearance pressures and body image ideals.

Given the importance of minority stress as a predictor of psychopathology, the high number of recently published studies and the potential health-related consequences of disordered eating, a systematic review is needed to summarize the latest evidence on these topics.

### Aims

The present article aims to summarize the scientific literature surveying the role of minority stress pertaining disordered eating in SGMs, with a particular focus on potential mediating factors, screening for any specificities and differences between the various identity categories.

Research questions include:

1. What are the effects of minority stress on disordered eating?
2. Are there any factors mediating the relationship between predictor and outcome?
3. Are there any specificities for SGM people regarding disordered eating?
4. Are there any differences between identity categories (e.g., lesbian, gay, bisexual, trans, gender non-conforming, nonbinary, etc.)?

## Methodology

### Review registration

The authors chose not to register the systematic review due to a lack of appropriate options in common registration services for the employed search framework.

### Information sources

This review employs the Preferred Reporting Items for Systematic Review and Meta-Analyses (PRISMA) statement [31]. Due to the observational nature of studies regarding the main topic, the Population, Exposure and Outcome (PEO) search framework [32] was used. Two independent reviewers (FS and LR) conducted a search through different academic search engines (EBSCO, PUBMED and Web of Science). Due to the high number of available records clearly meeting the exclusion criteria, several filters were applied to the search engines. Whenever possible, the search engines’ function of automatic duplicate removal was used.

EBSCO was filtered for academic articles across several different databases: APA PsycArticles, APA PsycInfo, CINAHL Complete, Education Source, Family Studies Abstracts, Food Science Source, FSTA—Food Science and Technology Abstracts, Gender Studies Database, Mental Measurements Yearbook, Sociology Source Ultimate, Violence and Abuse Abstracts.

PUBMED was filtered for the following categories: Abstract, Books and Documents, Case Reports, Classical Article, Clinical Study, Clinical Trial, Clinical Trial Protocol, Clinical Trial, Phase I, Clinical Trial, Phase II, Clinical Trial, Phase III, Clinical Trial, Phase IV, Comparative Study, Congress, Controlled Clinical Trial, Corrected and Republished Article, Evaluation Study, Meta-Analysis, Multicenter Study, Observational Study, Pragmatic Clinical Trial, Published Erratum, Randomized Controlled Trial, Retraction of Publication, Technical Report, Twin Study, Validation Study, Humans, English.

Web of Science was filtered for Articles. Due to limitations imposed by Web of Science on the number of search terms in the “all fields” type of string, search strings were limited to the following types: “all fields” (Population string); abstract (Exposure string); keywords (Outcome string).

No temporal limits were imposed on the search, which included articles from the beginning of the databases (1897) up to January 2024.

### Search strategy

To develop the search strings, the most relevant terms were selected and added to a singular field connected by “OR” operators. The “population” string was filled with terms relative to identification with sexual orientation or gender identity, as well as additional terms which may pertain to the relevant population despite being conceptualized differently (e.g., msm, wlw). The “exposure” string was filled with the main terms relevant to minority stress, covering both distal and proximal stressors; additionally, “psychological mediation” was added to cover any studies which may have employed the psychological mediation framework [26] without naming minority stress. The “outcome” string was filled with terms relevant to disordered eating (covering full-blown eating disorders and symptoms).

The following strings were used across three different fields connected by “AND” operators:

#### Population

“same-sex” OR “same-gender” OR gay OR lesbian* OR bisex* OR pansex* OR lgb* OR glb* OR blg* OR bgl* OR homosexual* OR “men who have sex with men” OR msm OR “women who have sex with women” OR wsw OR “men who have sex with men and women” OR msmw OR “women who have sex with women and men” OR wswm OR sexual minorit* OR gender minorit* OR “men who love men” OR mlm OR “women who love women” OR wlw OR trans* OR ftm OR mtf OR amab OR afab OR queer* OR “gender non-conforming” OR gnc OR nonbinary OR gender expansiv* OR agender* OR non-binary OR asexual* OR demisexual* OR gray-asexual* OR "gray-a" OR demisexual* OR intersex*

#### Exposure

“minority stress” OR minority stress* OR proximal stress* OR distal stress* OR "psychological mediation" OR internalized homophobia OR internalized biphobia OR internalized transphobia OR internalized lesbophobia OR heterosexis* OR internalized homonegativity OR stigma OR concealment OR discrimination OR expectation* of rejection

#### Outcome

Eating disorder* OR anorexia OR bulimia OR orthorexia OR avoidant restrictive food intake disorder OR disordered eating OR “binge eating” OR “food binge” OR overeat* OR undereat* OR diet* OR eat* OR underweight* OR exercis* OR purging OR “rumination regurgitation disorder*” OR thin ideal* OR muscular ideal*

## Inclusion and exclusion criteria

The following inclusion criteria were used to select studies during the screening phase:

- Original research paper.
- Published in peer reviewed journals.
- Written in the English language.

The following exclusion criteria were used to select or exclude studies during the screening and full-text review phases:

- Study does not analyze minority stress or disordered eating in its results.
- In defining its inclusion criteria for participants, study considers only participants’ sexual behavior (e.g., men who have sex with men) without including identity-related aspects (e.g., lesbian, gay, bisexual, trans, gender non-conforming).
- The measure used for analysis of minority stress does not specify identity-related aspects (e.g., general bullying with no references to SGM identity as a potential cause).

Ambiguous records that potentially met these criteria were selected for full-text review. Records that clearly did not meet these criteria (e.g., reviews of the literature, meta-analyses) were excluded during the screening phase.

### Study selection and data extraction

After automatic and manual duplicate removal, a total number of 2416 records were gathered across search engines for screening. Of these, 2351 were excluded due to inclusion and exclusion criteria. 65 reports were sought for retrieval. The two authors (FS and LR) independently screened the records, discussing inclusion and exclusion factors until unanimous consent was reached. The full text of the studies was then independently analyzed. After in-depth analysis and application of the inclusion and exclusion criteria, a final total of 30 studies were included in the systematic review.

**Fig. 1:**
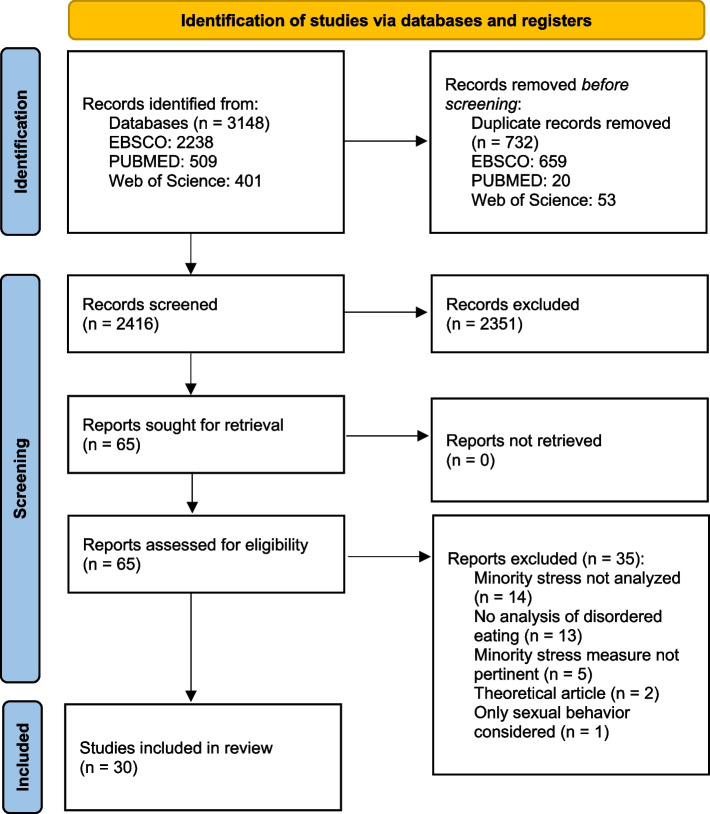
PRISMA Diagram—Identification of studies via databases and registers. A path diagram describing the review’s process for identifying studies. The process follows 3 phases (identification, screening and inclusion) and involves 5 stages. On the left side, the following stages are described: number of record identification in databases, number of records screened, number of records sought for retrieval, number of reports assessed for eligibility, and finally, number of studies included in the review. Each of the initial 4 stages on the left side has a corresponding stage on the right side where numbers and reasons of removal or exclusion are detailed

Figure 1 summarizes the systematic review process as per the PRISMA statement [31].

### Risk of bias assessment

Risk of bias was assessed using the JBI suite of Critical Appraisal Tools [33, 34]. For most of the studies, the Analytical Cross-Sectional Studies checklist [35] was employed. For one study [36], the Qualitative Research checklist [37] was employed. For another study [38], the Cohort Studies checklist [35] was employed. Exposure was conceptualized to be relative to minority stress factors, while outcomes were conceptualized to be relative to disordered eating factors. Assessment was independently carried out by two evaluators. Evaluations were discussed until unanimous consent was reached.

## Results

### Synthesis of results

Online Resource 1 synthesizes the included studies’ main characteristics and their most relevant results. For each included study, data analysis methods, employed measures, relevant variables, as well as significant and nonsignificant relationships were identified and synthesized.

### Studies’ characteristics

#### Design

Almost all the included studies adopted a cross-sectional design. Only one study [38] conducted a longitudinal cohort study. Another [39] employed a daily diary approach, analyzing its data as cross-sectional.

#### Measures

Almost all the included studies involved analyzing quantitative data from a structured questionnaire consisting of previously validated measures. Additionally, for data collection of some variables, seven studies [10, 40–45] administered questions that were developed outside a validated measure (ad hoc) either in the same study or in a previous study. Only one study [36] included qualitative analysis of open-ended questions (reflexive thematic analysis).

#### Risk of bias

Several domains of possible bias were identified. In about two thirds of the studies, study participants were not described in-depth. Most commonly, information about education, socio-economic status and time of participation was missing, which may lead to issues in assessing the participants’ comparability relative to other samples. Identification and control of possible confounding factors was missing or partial in about a third of the studies, which may influence the certainty or reproducibility of their findings. Finally, a small minority of studies showed possible issues in their measurement of exposure or outcomes, either by using ad hoc measures or by adapting measures meant for more general discrimination.

In-depth evaluations are available in Online Resource 2, which provides study-level assessments.

### Disordered eating in SGMs

#### Effects of minority stress^2^

Regarding distal stressors, experiences of discrimination were associated with disordered eating [46–48], eating disorder pathology [49] and binge eating [50], except for one study [39]. Anti-bisexual discrimination was associated with disordered eating [51] and emotional eating [41]. Heterosexist discrimination was associated with higher odds of eating pathology [52] and food addiction symptoms [53], except for one study regarding dysregulated eating [43]. Enacted stigma was associated with binge eating, fasting, and vomiting to lose weight [45]. Antitransgender discrimination [11, 46], rejection, victimization and non-affirmation [46] were associated with disordered eating.

Regarding proximal stressors, internalized homophobia was associated with disordered eating [48, 54], higher odds of eating pathology [52] and binge eating [43, 55]. However, in several studies [10, 48, 56, 57], the association between internalized heterosexism/homophobia and disordered eating was not found. Another study found the association regarding subjective (but not objective) binge eating, and in addition, this relationship was not moderated by distress tolerance [55]. One study found internalized cisgenderism predicted higher levels of disordered eating [58]. Sexual orientation concealment was associated with disordered eating [48, 54], higher odds of eating pathology [52] and overeating [43]. Stigma consciousness was associated with higher odds of overeating [10]. Internalized transphobia, negative expectations and gender identity concealment were associated with disordered eating [46].

#### Mediating factors

Regarding distal stressors, the relationship between antibisexual discrimination and disordered eating was partially mediated by internalization of cultural standards of appearance, body surveillance and body shame [59], as well as internalization-based coping [51]. Body surveillance and body shame also mediated the relationship between internalized heterosexism and negative eating attitudes [56]. The relationship between discrimination and binge eating was mediated by social anxiety and body shame [50] as well as negative affect and self-awareness [39].

Regarding proximal stressors, the relationship between proximal stressors and binge eating behaviors was mediated by social isolation, emotion-focused coping and negative affect [42]. Shame mediated the relationship between internalized homophobia and subjective binge eating, an aspect which again was not moderated by distress tolerance [55]. In another study, for men only, rumination mediated the paths from discrimination and internalized homonegativity to disordered eating [48]. In another study, body shame mediated the relationship between internalized cisgenderism and disordered eating [58].

In trans people, partial mediation of eating-disorder-specific rumination was found between gender minority stress and eating disorder psychopathology [60]. Another study [61] found gender dysphoria fully mediated the relationship between minority stress and eating pathology. Yet another found internalized transphobia mediated between discrimination trauma and eating pathology [62].

Regarding mediation as well, some associations were found to be nonsignificant. Occasionally, paths from distal to proximal minority stress [42, 57] and from proximal minority stress to binge eating [42] were nonsignificant. In another study, the indirect associations between internalized biphobia and eating disorder symptoms were nonsignificant [59]. Depression additionally did not mediate the relationship between internalized homophobia and binge eating [55].

#### Specificities of SGM people

Several studies found a role of community involvement. One pair of studies drawing from the same sample found an association between LGBTQIA + community involvement and eating pathology [52, 63], additionally finding interaction effects between community involvement and heterosexist discrimination on internalized homophobia and sexual orientation concealment. Another, however, found a negative correlation between community connectedness and disordered eating [46]. One study, instead, found no such correlation with eating disorders [49].

In this regard as well, trans people showed some specificities. One study highlighted how gender minority stress had an effect on eating disorder psychopathology, and was associated with gender-related rumination and eating-disorder-specific rumination [60]. Another found a connection between gender minority stressors, internalized transphobia and disordered eating [46]. Furthermore, another study showed how higher transgender congruence (i.e., perceived congruence between one’s gender identity and gender expression), past-year gender-affirming therapy and internalized transphobia were associated with lower odds of eating-related psychopathology [38]. The role of gender dysphoria in predicting eating pathology was highlighted as well [61, 62], which in addition highlighted associations to anxiety and depression [61].

One study found the stress of coming out was associated with higher odds of clinical threshold caloric restriction, purging and binge eating [44].

Finally, one study found positive feelings about one’s SGM identity were associated with lower odds of clinical threshold caloric restriction, purging and binge eating, while openness about one’s SGM identity was associated with lower odds of clinical threshold caloric restriction [44]. Another, however, contrary to the authors’ expectations, found a moderate connection between pride in one’s gender identity and disordered eating [46].

#### Differences between groups

For women, prevalence of probable eating disorder diagnosis was notably higher for bisexual women (23,4%) than lesbian women (10%) [47]. Two studies additionally found bisexual people had higher levels of internalized homophobia compared to lesbian women [10] and gay men [40]. However, others found no differences in the likelihood of binge eating [42, 55], compensatory behaviors or internalized homophobia [55] when drawing the same comparison between bisexual and lesbian women.

Furthermore, some differences between men and women were found. In one study, main effects of community involvement on sexual orientation concealment and internalized homophobia were significant for men but not women [63]. In a study conducted by Wang and colleagues [48], rumination did not mediate between concealment and disordered eating for women, and additionally the relationship between internalized negativity and concealment was significant for men but not women.

Finally, one study on SGM adolescents found gender minorities exhibited a higher prevalence of caloric restriction, purging and binge eating when compared to sexual minorities [44].

## Discussion

### Critical discussion of evidence

The included literature features some methodological criticalities that should be kept in mind while interpreting its results. As a whole, it is very homogeneous regarding the use of cross-sectional designs and quantitative approaches. This results in an overwhelming lack of qualitative studies, which may create a blind spot regarding the internal psychological experiences of participants, as well as a lack of longitudinal approaches, which could have drawn attention to how the surveyed relationships evolve in time and given further empirical support to the hypothesized relationships. Furthermore, the risk of bias evaluation showed not all of the included studies described attempts to control for possible confounding factors (e.g., body-mass index [BMI] or age)—a limitation which may possibly affect the certainty of their findings. The occasional use of ad hoc measures should also warrant caution in the generalizability of the relevant findings.

Coherently with previous available evidence [9, 23, 25, 64], minority stressors appear to be consistently associated with worse disordered eating outcomes, often resulting in moderately strong associations and explaining good amounts of variance in the models in which they are present. Overall, the presently included literature appears to support the importance of minority stress as a predictor of disordered eating behaviors. Some conflicting findings remain regarding the significance of links between some distal (i.e., discrimination) or proximal stressors (i.e., internalized heterosexism, homonegativity, or homophobia) and specific disordered eating behaviors; however, the respective authors interpret some nonsignificant findings to be due to statistical power (e.g., low levels of heterosexism as in [57]; low sample sizes as in [39, 55]). Notably, studies with far larger sample sizes (e.g., [52, 54]) all found the same relationships to be statistically significant. The included studies also provide several insights regarding how minority stressors determine heightened risk outcomes, describing their effects on general psychological processes mediating these relationships, including intrapsychic (e.g., negative affect, general shame, and body shame) and social (e.g., internalization of sociocultural standards of appearance, social anxiety, social isolation) factors. The roles of negative affect and shame appear particularly salient, as these have been reliably linked to eating disorders [65].

The surveyed literature mostly confirms for SGMs some aspects that apply to the general population. Save for some exceptions [11, 46], age and BMI (despite the persistence of criticisms on its usage as a measure) have been found to have a connection with body image—specifically, a younger age [40, 58] and a higher BMI [41, 50, 53, 58, 66] were associated with worse disordered eating behaviors.

According to the results, the minority stress model also seems to mostly integrate with other theoretical frameworks [54, 59, 63], such as the Tripartite Influence Model [15, 16] or Objectification Theory [17, 18], explaining unique additional variance when taken into account. These contributions further add to the understanding of the complex interconnections involved in disordered eating.

The available evidence also highlights some similarities regarding the existence of appearance ideals, such as those based on thinness and muscularity [54, 63], while also suggesting divergences, such as the contemporaneous existence of ideals based on both thinness and muscularity in gay men [63].

The role of LGBTQIA + communities appears to be important, but ambivalent [46, 52, 63]. Contrary to general minority stress theories, higher involvement with LGBTQIA + appears to sometimes promote a higher risk of disordered eating behaviors, a finding whose interpretation is complex. Qualitative [67] evidence appears to point to additional appearance pressures felt from communities, a finding which may work in tandem with internal community competitiveness [68]. These findings, however, should be interpreted with caution and explored further in research, as generalizations may be misleading given the heterogeneity of LGBTQIA + communities, especially on an international level.

Bisexual people appear to be at particularly high risk regarding disordered eating. These outcomes could stem from being exposed to multiple discrimination sources [69, 70], both from heterosexual people (e.g., for being attracted to people of the same gender) and gay/lesbian people, due to stereotypical beliefs and prejudices about bisexuality (e.g., identity instability, sexual promiscuousness and irresponsibility) and for being attracted to people of other genders.

Gender minorities also show several specificities. In addition to showing higher risk compared to sexual minorities [44], the existence of gender-stereotypical links between thinness, femininity, muscularity and masculinity [36] appears to provide an additional drive for trans people regarding the attainment of thinness or muscularity—attainment of congruence between one’s gender identity and the perceived body image ideal [11, 59]. Disordered eating behaviors may therefore be engaged in order to attain the appearance ideal related to one’s gender identity [59, 71]. Additionally, antitransgender discrimination and violence experiences in trans people [11, 71] may be interpreted to relate to perceived gender congruence (i.e., “I am being discriminated because I am not passing”), and further drive disordered eating behaviors.

Among the included studies, some identities appeared present but underrepresented in analyses: few studies [36, 38, 43, 46, 60–62] reported explicitly on gender non-conforming and nonbinary people. These identities were also sometimes grouped together with other gender minorities (e.g., trans people) or under other umbrella terms (e.g., queer). The same can be said about asexual people, which were sometimes included [11, 36, 48] but never explored in particular. As discussed by other authors [30, 72], if at all possible, it may be ideal to integrate analyses screening for differences and treat every identity as unique, as grouping different categories may create other blind spots regarding one’s experiences, as was the case for gendered conceptions of body ideals in gender minorities.

Finally, while some interactions between sexual orientation and gender identity were highlighted in the included studies, another understudied aspect relates to how minority stress interacts with other aspects of identity, such as race and class, which may offer an intersectional perspective [73]. This appears to be a common issue in literature [24, 73], where an intersectional perspective is often limited to studies whose focus is explicitly on intersectional analyses rather than being integrated in studies surveying other aspects. As highlighted by this review’s risk of bias evaluation, several of the included studies did not provide complete sociodemographic information, which may additionally prove limiting when trying to compare results across populations. Many of the included studies collected data about racial and ethnical identity or socio-economic status, but seldom used it as an additional lens, with some notable exceptions including racial identity as a variable [38, 40, 74, 75]. Additional quantitative evidence shows an interaction between race/ethnicity and sexual orientation [76]. The same could be said about socio-economic status, which has also been found to determine a higher risk of disordered eating [76, 77] through its influence on, among other factors, food insecurity and emotional regulation. Considering the role of weight status in disordered eating and the findings regarding weight bias in SGMs [12–14], intersectional analyses involving weight status may also be beneficial.

### Strengths and limits

This review synthesizes a large amount of evidence on a complex topic and is set up as a replicable and updatable protocol. Due to the high number of search terms and engines employed, it is likely to have covered a large part of the available literature that respects its inclusion and exclusion criteria.

Some methodological limitations should be kept in mind while evaluating the emerging themes and results. The current systematic review of the literature features no meta-analysis, and as such cannot be used to derive generalized statistical conclusions on the collected evidence. Additionally, it only includes articles written in the English language. By excluding evidence that is only available in other languages, results may be biased by culturally homogeneous environments and potentially create a blind spot for cultural and international differences. Finally, the employed methodology is only able to detect a limited subset of all available literature (e.g., due to the choice of inclusion criteria, search engines, databases, and search terms), and as such this review should not be inferred to be completely comprehensive of all the existing relevant literature.

### Future directions

The most glaring gap in the surveyed literature regards longitudinal studies, which are extremely rare—more longitudinal studies may help build more confidence in the identified relationships. The same can be said for qualitative approaches: conducting more qualitative studies may help gain a better focus on individuals’ specific experiences.

The risk of bias evaluation also highlighted some potential issues in the included literature. Future studies should attempt to identify and control for possible confounding factors, include more complete sociodemographic information, and possibly use standardized measures. While the role of many social factors has been extensively studied, data on some intrapsychic factors (e.g., emotion regulation) remains limited. More studies involving these factors could prove beneficial to understanding internal psychological mechanisms that lead to heightened risks, which may provide useful clinical insights.

Integrating intersectional perspectives that screen for interaction effects of one’s various facets of identity may prove beneficial in more comprehensively individuals’ specificities, especially when considering multiply marginalized identities. This may offer unique perspectives to explain how SGM minority stressors interact. Cross-cultural and LGBTQIA + -specific comparisons, which were absent, may also add to these perspectives.

Finally, gender non-conforming, nonbinary, and asexual people appear to be understudied populations in this field which may benefit from additional focus.

## Conclusions

Minority stress constitutes an important predictor for additional health risks regarding disordered eating, acting upon several psychological and social processes. The implementation of anti-discrimination policies/interventions and LGBTQIA + -specific cultural competencies in clinical, institutional, and organizational settings may provide improvements for physical and mental health benefits.

### What is already known about this subject?

Risk of disordered eating behaviors is higher in SGMs compared to heterosexual and cisgender people, with significant impacts on their physical and mental health. A large amount of evidence highlights minority stress as a possible explanation for this disparity, with some ambivalent aspects. No synthesis is available as to what its role is, what mediates its effects, and whether any specificities or differences for identity categories are present.

### What does this study add?

The review offers a more nuanced overview of how minority stress affects SGMs in the context of disordered eating. Minority stressors determine disparities in psychological health by affecting several psychosocial aspects. Specificities and differences are present regarding gender identity and sexual orientation.

### Supplementary Information

Below is the link to the electronic supplementary material.

Supplementary file1 (XLSX 18 KB)

Supplementary file2 (PDF 368 KB)

## Data Availability

Not applicable.

## Footnotes

1 For disambiguation of SGM-related terms, readers may refer to the APA’s dictionary of psychology [4] or the INSIGHT Project’s Good Practices Guide glossary [5].

2 All associations and correlations are intended to be positive unless otherwise stated.
