# Supplementary material for: The role of minority stress in disordered eating: a systematic review of the literature
Source: Eat Weight Disord. 2024 Jun 8;29(1):41. doi: 10.1007/s40519-024-01671-7 (PMC11162380; doi:10.1007/s40519-024-01671-7)
Supplement: Supplementary file 2 — Supplementary file2 (PDF 368 KB) [file 40519_2024_1671_MOESM2_ESM.pdf]

Article Title: The role of Minority Stress in Disordered Eating – A Systematic Review of the Literature

Journal Name: Eating and Weight Disorders - Studies on Anorexia, Bulimia and Obesity

Authors: Fabrizio Santoniccolo* (0000-0003-4475-5539), Luca Rollè (0000-0002-4398-8683)

Affiliation: Department of Psychology, University of Turin, via Verdi 10, 10124, Torino (TO), Italy

***Corresponding author email**: fabrizio.santoniccolo@unito.it

Study	Participants	Data analysis	Variables	Measures	Significant Results[1]	Nonsignificant Results
Haines et al., 2008	150 lesbian women (mean age 35.12 years)	Bivariate correlations, path analysis	internalized heterosexism, feminist self-identification, negative eating attitudes, depressive symptomatology, body surveillance, body shame	Lesbian Internalized Homophobia Scale<br>Objectified Body Consciousness Scale<br>Eating Attitudes Test<br>Center for Epidemiologic Studies Depression Scale	**Prevalence**<br>25,3% of the sample was above the clinical cut-off for depressive symptomatology; 12% of the sample scored above the clinical cut-off for negative eating attitudes.<br>**Bivariate correlations**<br>Internalized heterosexism was negatively correlated with feminist self-identification, positively correlated with surveillance, body shame and depression.<br>**Path model**<br>Model had good fit to the data, explaining 30% of the variability in negative eating attitudes scores and 29% of depression scores. All paths but one were significant. Body surveillance and body shame mediated the relationship between internalized heterosexism and negative eating attitudes, as well as depressive symptomatology.	**Bivariate correlations**<br>Feminist self-identification was not related to self-objectification variables or clinical outcomes. Internalized heterosexism was not related to negative eating attitudes.<br>**Path model**<br>Feminist self-identification path to body surveillance was non-significant.
Brennan et al., 2012	400 men (350 gay, 34 bisexual) aged 16-76	Bivariate correlations, multivariate analyses, regression analyses	drive for muscularity, age, race, education, disordered eating behaviors, HIV status, internalized homophobia, substance use	Ad-hoc questions for sexual behavior, sexual abuse, and HIV status<br>Center for Epidemiologic Studies Short Depression Scale<br>Internalized Homonegativity Scale<br>Eating Attitudes Test<br>Drive for Muscularity Scale	**Prevalence**<br>14% of the participants were at high risk of disordered eating symptomatology<br>**Bivariate correlations**<br>Drive for muscularity was positively associated with disordered eating behaviors.<br>**Multivariate analyses**<br>Participants reporting high internalized homonegativity reported a higher drive for muscularity than those reporting low internalized homonegativity.<br>Participants over 50 years old had lower drive for muscularity compared to participants of other age groups.<br>**Regression analyses**<br>Predictors accounted for 10% in the variance in drive for muscularity.<br>Having high risk for disordered eating symptomatology, high levels of internalized homonegativity, higher levels of depression and being diagnosed with an STI predicted a higher drive for muscularity.<br>Being over 50 years old predicted a lower drive for muscularity compared to the 16-25 years old age group.	N/A
Brewster et al., 2014	316 bisexual women aged 18-69 years old (93% women, 2% trans women, 5% other)	Path analysis	internalized biphobia, sexual objectification, anti-bisexual discrimination, internalization of Cultural	Anti-bisexual Experiences Scale<br>Internalized Homonegativity subscale of the Lesbian, Gay, Bisexual and Identity Scale<br>Interpersonal Sexual	**Direct relationships:** Antibisexual discrimination showed significant positive direct relation with internalization of Cultural Standards of Appearance (CSA) and internalized biphobia. Internalization of CSA showed significant positive direct relation with body surveillance. Antibisexual discrimination, internalization of CSA, and body surveillance showed significant direct relations with body shame. Sexual objectification, internalization of CSA and body shame showed significant direct relations with eating disorder symptoms. **Indirect relationships:** Internalization of CSA	Internalized biphobia did not show any total or unique indirect links with eating disorder symptoms or body shame.

[1] All reported associations and correlations statistically significant unless otherwise stated.

			Standards of Appearance (CSA), body surveillance, body shame, eating disorder symptoms	Objectification Scale<br>Internalization subscale of Sociocultural Attitudes Towards Appearance Questionnaire<br>Body Surveillance and Body Shame subscales of the Objectified Body Consciousness Scale<br>Eating Attitudes Test-26	showed an indirect link with body shame through body surveillance. Body surveillance showed an indirect link with eating disorder symptoms through body shame. Internalization of CSA showed an indirect link with eating disorder symptoms through body shame and body surveillance. Antibisexual discrimination showed an indirect link with body surveillance through internalization of CSA. Antibisexual discrimination showed an indirect link with eating disorder symptoms through internalization of CSA, body surveillance and body shame. Indirect links of antibisexual discrimination with body surveillance and eating disorder symptoms reflect full mediation, while indirect links with body shame reflect partial mediation.	
Mason & Lewis, 2015	164 lesbian and bisexual women aged 18-40 who did not engage in any compensatory behaviors indicative of bulimia nervosa	Structural Equation Modeling	Minority stress (distal & proximal), Social Isolation, Emotion-Focused Coping, Negative Affect, Binge Eating	Internalized Homophobia Scale - Revised<br>Stigma Consciousness Questionnaire<br>Ad-hoc single item for sexual orientation openness<br>Heterosexist Harassment, Rejection and Discrimination Scale<br>Friendship Scale<br>Cognitive Emotion Regulation Questionnaire<br>Mental Health Inventory<br>Binge Eating Scale	**Prevalence**<br>13,4% of participants engaged in moderate binge eating, 4,9% in severe binge eating.<br><br>**Structural Equation Modeling**<br>Proximal minority stressors were indirectly related to binge eating behaviors through social isolation and negative affect. Proximal minority stressors were indirectly related to binge eating behaviors through emotion-focused coping and negative affect.	Paths from social isolation to negative affect, from distal to proximal minority stress and from proximal minority stress to binge eating were nonsignificant.
Watson et al., 2015	234 sexual minority women (18+); 107 identified as lesbian, 63 as bisexual, 31 as queer, 28 as pan, 9 as questioning, 2 as omnisexual, 3 as another unlisted identity	Path analysis	Heterosexist events, internalized heterosexism, sexual objectification experiences, internalized sociocultural standards of beauty, body surveillance, body shame, disordered eating	Interpersonal Sexual Objectification Scale<br>Sociocultural Attitudes Towards Appearance Questionnaire - Internalization Subscale<br>Objectified Body Consciousness Scale - Body Surveillance & Body Shame Subscales<br>Eating Attitudes Test-26<br>Heterosexist Harassment, Rejection and Discrimination Scale<br>Lesbian Internalized Homophobia Scale	**Correlations**<br>Most variables had significant positive correlations between each other.<br><br>**Direct effects**<br>Most paths from the hypothesized model were significant: Internalized sociocultural standards of beauty to body surveillance to body shame to disordered eating; Sexual objectification experiences to internalized sociocultural standards of beauty; Internalized heterosexism to body surveillance and body shame.<br><br>**Indirect effects**<br>Internalized sociocultural standards of beauty fully mediated the relationship from sexual objectification experiences to body surveillance. Internalized sociocultural standards of beauty also fully mediated the relationships from sexual objectification experiences to body shame and disordered eating. Body shame fully mediated the relationship between body surveillance and disordered eating symptoms.	**Correlations:** Experiences of heterosexism were not related to internalized heterosexism. Internalized heterosexism was not related to sexual objectification experiences.<br>Sexual objectification was not related with body shame.<br><br>**Direct effects:** Paths from sexual objectification experiences to body surveillance; from body surveillance to disordered eating; from internalized heterosexism to disordered eating; from heterosexist experiences to internalized sociocultural standards of beauty, internalized heterosexism, body surveillance and body shame.<br><br>**Indirect effects:** Body surveillance did not mediate the relationship between heterosexist discrimination and body shame, and neither between sexual

						objectification experiences and body shame.
Mason & Lewis, 2016	496 lesbians (18-30 years-old, self-identified)	Structural Equation Modeling	Discrimination, proximal minority stress (Acceptance concerns, Concealment motivation, Difficult process, internalized homophobia), Social anxiety, Body shame, Binge eating, BMI	Everyday Discrimination Scale<br>Lesbian, Gay and Bisexual Identity Scale<br>State Social Anxiety Questionnaire<br>Objectified Body Consciousness Scale - Body Shame subscale<br>Eating Disorder Inventory - Bulimia subscale and Eating Disorder Diagnostic Scale	**Correlations**<br>All study variables were correlated with body shame and binge eating.<br>**Direct effects**<br>Experiences of discrimination were associated with more proximal minority stress and social anxiety.<br>Proximal minority stress was associated with social anxiety and body shame. Social anxiety was associated with increased with body shame and binge eating. Body shame was associated with binge eating. BMI was associated with body shame and binge eating.<br>**Indirect effects**<br>Four pathways to binge eating were found. Discrimination was associated with binge eating via proximal minority stress, social anxiety, and body shame; minority stress and social anxiety; minority stress and body shame; social anxiety only.	Path from Discrimination to BMI
Watson et al., 2016	353 bisexual women 18 to 61 years old	Structural Equation Modeling	Anti-bisexual discrimination, disordered eating, outness, maladaptive coping (internalization, detachment, drug and alcohol use)	Anti-bisexual Experiences Scale<br>Outness Inventory, adding an additional item for openness with new lesbian/gay friends.<br>Coping with Discrimination Scale - Internalization, Detachment and Drug and Alcohol Use subscales - Eating Attitudes Test-26	**Bivariate correlations**<br>Anti-bisexual discrimination had small positive correlations with internalization, detachment, drug and alcohol use, and disordered eating.<br>Outness yielded a small negative correlation with disordered eating.<br>**Latent variable correlations**<br>Mostly consistent with bivariate correlations.<br>**Structural equation model**<br>The structural model provided acceptable fit, accounting for 21% of the variance in disordered eating, 20% in outness, 14% in detachment, 7% in drug and alcohol use, and 6% in internalization.<br>**Direct effects:**<br>Anti-bisexual discrimination had positive unique relationships with internalization, detachment, drug and alcohol use and disordered eating.<br>Internalization yielded a positive unique relationship with disordered eating.<br>**Indirect effects:**<br>Anti-bisexual discrimination had a unique positive indirect relationship with disordered eating through internalization.	**Bivariate correlations:**<br>Anti-bisexual discrimination was not related with outness.<br>**Latent variable correlations:**<br>Anti-bisexual discrimination was not related with outness.<br>Outness was not related with drug and alcohol use.<br>**Structural equation model**<br>**Direct effects:**<br>Anti-bisexual discrimination was not related to outness.<br>The negative association between outness and disordered eating approached but did not reach significance.<br>Detachment, as well as drug and alcohol use, were not related to disordered eating.<br>**Indirect effects:**<br>Outness, detachment, as well as drug and alcohol use did not mediate the association between anti-bisexual discrimination and disordered eating.
Bayer et al., 2017	A total of 138 lesbian (n = 72) and bisexual (n = 66) women. Age differences not significant.	Mediation analysis	Objective and subjective binge eating, internalized homophobia, shame,	Eating Disorder Examination Questionnaire - items about binge eating only, in addition to a single question for purging behavior frequency<br>Lesbian Internalized	**Bivariate correlations**<br>All variables were correlated with each other.<br>**Mediation analysis**<br>**Direct effects:**<br>Internalized homophobia had a direct effect on subjective binge eating.<br>**Indirect effects:**	**Mediation analysis**<br>**Direct effects:**<br>Internalized homophobia did not have a direct effect on objective binge eating.<br>**Indirect effects:**<br>Internalized homophobia did not

			depression, distress tolerance	Homophobia Scale - Personal Feelings about Being Lesbian/Bisexual subscale<br>Personal Feelings Questionnaire - Shame and filler items subscales<br>Depression, Anxiety and Stress Scale - Depression subscale<br>Distress Tolerance Scale	Internalized homophobia had an indirect effect on objective and subjective binge eating through shame.	have an indirect effect on objective or subjective binge eating through depression.<br>Distress tolerance did not moderate relationships between internalized homophobia and binge eating or between shame and binge eating.
Mason et al., 2017	30 lesbian women aged 18-30 that had at least one binge eating episode (defined as both consuming a large amount of food in a short time and experiencing loss of control).	1-1-1 Multilevel Structural Equation Modeling	Perceived discrimination, negative affect, self-awareness, binge eating	Everyday Discrimination Scale - modified to specify gender-based and sexual orientation-based discrimination for each item<br>Positive and Negative Affect Schedule - Negative affect items only.<br>Mindful Attention Awareness Scale<br>Eating Disorder Inventory - Bulimia and Eating Disorder Diagnostic subscales, reduced to their behavioral components.	**Path analysis**<br>Discrimination was associated with negative affect and self-awareness.<br>Negative affect was associated with self-awareness.<br>Self-awareness was associated with binge eating.	**Path analysis**<br>Discrimination was only marginally associated with binge eating ($p = .10$).<br>Path from negative affect to binge eating was nonsignificant.
Wang & Borders, 2017	116 sexual minority individuals aged 18 to 40, article focuses on the male 59.5%	Path analysis	Sexual minority discrimination, internalized homonegativity, sexual orientation concealment, rumination, disordered eating	Everyday Discrimination Scale (EDS)<br>Lesbian, Gay and Bisexual Identity Scale (LGBIS) - Internalized Homonegativity and Concealment Motivation subscales<br>Interpersonal Offences Scale (IOS) - modified by the authors to assess rumination about perceived sexual minority stigma<br>Eating Attitudes Test-26 (EAT-26).	**Correlations**<br>Greater discrimination, internalized homonegativity and concealment were correlated with more rumination and disordered eating.<br>**Path analysis**<br>Two models were tested: unconstrained multi-group, multi-group with all coefficients constrained across gender. Both were just-identified. The first model had an excellent fit. The second model had a poor and significantly worse fit. Authors assume coefficients for sexual minority men and women not to be the same.<br>**Total effects**<br>In the unconstrained model, discrimination and concealment predicted disordered eating.<br>For men but not women, discrimination and concealment were related.<br>**Mediation analyses**<br>A mediation model was created that used rumination as mediator from discrimination, internalized homonegativity and concealment to disordered eating.<br>Direct effects of discrimination and concealment were significant.<br>Rumination mediated the paths from discrimination and concealment to disordered eating.<br>For men only, discrimination and concealment predicted rumination and rumination predicted disordered eating.<br>For women, discrimination predicted rumination.	**Path analysis**<br>**Total effects**<br>In the unconstrained model, internalized homonegativity did not predict disordered eating.<br>**Mediation analyses**<br>The direct and indirect effects of internalized homonegativity did not predict disordered eating.<br>For women only, concealment and internalized homonegativity did not predict rumination.
Watson et al., 2017	923 transgender youth aged 14-25 years old	Logistic regressions;	Transgender identity, enacted stigma, school connectedness,	Single-item transgender identification question - options meant to categorize transgender girls/women,	**Prevalence**<br>46% of participants reported no disordered eating behavior. 26% reported one disordered eating behavior.<br>42% of 14–18-year-old participants reported binge eating at least once during the	**Bivariate/multivariate correlations**

		Probability profiling	family connectedness, perception of friends caring, social support, binge eating, purging	boys/men, non-binary<br>Enacted Stigma Index<br>Ad-hoc questions for school connectedness, family connectedness (14-18 year olds)<br>Minnesota Student Survey, single item for measuring perception of friends caring (14-18 year olds)<br>Modified version of the Medical Outcomes Study Social Support Survey (19-25 year olds)<br>Single ad-hoc item for binge eating<br>Single ad-hoc item for fasting, diet pills, laxatives and vomiting	last 12 months. Weight-loss behaviors ranged from 48% (fasting) to 5% (laxatives). 29% of 19–25-year-old participants reported the same. Weight-loss behaviors ranged from 27% (fasting) to 3% (laxatives).<br>**Bivariate/multivariate correlations**<br>Enacted stigma experiences were positively associated with binge eating, fasting, vomiting to lose weight.<br>School connectedness, family connectedness, perception of friends caring, and social support were inversely related with binge eating, fasting, vomiting to lose weight.	Stigma was not associated with losing weight using laxatives.
Rainey et al., 2018	Total of 356 participants (231 in round 1, 166 in round 2), of which 135 were sexual minority. Sample was 56.7% heterosexual, 22,5% lesbian or gay, 20.8% bisexual.	Correlational analyses, linear regressions	Food addiction, heterosexist discrimination, self-compassion, sexual orientation, age, BMI	Yale Food Addiction Scale<br>Heterosexist Harassment, Rejection and Discrimination Scale<br>Self-compassion Scale	**Correlational analyses:**<br>In the whole-group analysis, age and BMI were associated with food addiction symptom count and included as covariates.<br>In the sexual minority group analysis, BMI was associated with symptom count and was included as covariate.<br>**Linear regressions:**<br>Sexual orientation was associated with symptom count: sexual minority people were endorsing more symptoms than heterosexual people. The association remained significant when controlling for age and BMI.<br>BMI was associated with meeting the threshold for a food addiction diagnosis.<br>Within the sexual minority group, less self-compassion and more heterosexist discrimination were associated with more food addiction symptoms, and the associations remained significant when controlling for BMI.	**Correlational analyses:**<br>In the whole-group analysis, sex and ethnicity were not associated with symptom count.<br>In the sexual minority group analysis, sex, age, and ethnicity were not associated with symptom count.<br>**Linear regressions:**<br>Sexual orientation was only associated with food addiction without controlling for age and BMI. When controlling, this relationship became nonsignificant.<br>In the sexual minority group analysis, self-compassion was marginally associated (p=.052) with the threshold for food addiction diagnosis, while the relationship with heterosexist discrimination was not significant.
Brewster et al., 2019	205 transgender women aged 18-68 years old (mean 31.22) from the United States. Approximately 21% identified as exclusively lesbian, 21% as bisexual, 14% as mostly lesbian, 13%	Structural Equation Modeling	Antitransgender discrimination, sexual objectification experiences, internalization of sociocultural standards of appearance, body surveillance, body	Heterosexist Harassment, Rejection and Discrimination Scale modified for use with transgender people<br>Interpersonal Sexual Objectification Scale<br>Sociocultural Attitudes Toward Attractiveness Questionnaire-3 - Internalization subscale	**Structural Equation Modeling**<br>The measurement and structural models both yielded acceptable fit to the data. The hypothesized structural model accounted for 12% of the variance in internalization, 14% in body surveillance, 32% in body dissatisfaction, and 71% in disordered eating.<br>**Latent variables' correlations**<br>Dehumanization yielded small to large correlations with body surveillance, body dissatisfaction.<br>Body surveillance yielded a large positive correlation with body dissatisfaction.<br>Body satisfaction yielded a large positive correlation with disordered eating.	**Structural Equation Model**<br>**Latent variables' correlations**<br>Body surveillance had no significant correlation with dehumanization or disordered eating.<br>Age had no significant correlation with internalization and disordered eating.<br>BMI did not yield significant correlations with any variable.

	as pansexual, 9% as heterosexual, 8% as some other sexual orientation (e.g., demisexual, asexual), 7% as queer, and 7% as mostly heterosexual.		dissatisfaction, disordered eating	Objectified Body Consciousness Scale - Body surveillance subscale<br>Body Image Ideals Questionnaire<br>Eating Attitudes Test-26	Age yielded small to medium negative correlations with dehumanization, body surveillance and body dissatisfaction.<br>**Structural model**<br>**Direct effects:**<br>Dehumanization yielded positive unique relations with internalization and disordered eating.<br>Internalization yielded positive unique relations with body surveillance, body dissatisfaction and disordered eating.<br>Contrary to expectations, body surveillance had a negative unique relation with disordered eating.<br>**Indirect effects:**<br>Dehumanization yielded a positive total relation with disordered eating through internalization, internalization and body surveillance (negative relation, contrary to expectations), and internalization and body dissatisfaction.<br>Dehumanization yielded a positive indirect relation to body dissatisfaction through internalization.<br>Internalization yielded a total positive indirect relation with disordered eating, primarily through body dissatisfaction.<br>Contrary to expectation, internalization yielded a negative indirect relation with disordered eating through body surveillance.	**Structural model**<br>**Direct effects:**<br>Body surveillance yielded a marginally significant (p=.05) unique relation with body dissatisfaction.<br>Relations from dehumanization to body surveillance and body dissatisfaction were nonsignificant.<br>**Indirect effects:**<br>Total indirect relation from dehumanization to body dissatisfaction was nonsignificant.<br>Indirect relation from internalization to body dissatisfaction was nonsignificant.<br>Indirect relation from body surveillance to disordered eating through body dissatisfaction was nonsignificant.
Mason & Lewis, 2019	436 cisgender lesbian women (self-identified) aged 18-30 (mean = 21.97) from the United States	Latent profile analysis	Disordered eating, minority stress, alcohol use, physical activity, negative affect, social anxiety, social support, discrimination, BMI	SCOFF<br>Eating Disorder Inventory–Bulimia Scale - 5 items for binge eating<br>Eating Disorder Diagnostic Scale - 7 items for binge eating<br>Alcohol Use Disorders Identification Test<br>International Physical Activity Questionnaire<br>Short Positive and Negative Affect Schedule<br>State Social Anxiety Questionnaire - modified<br>Multidimensional Scale of Perceived Social Support<br>Lesbian, Gay, and Bisexual Identity Scale	A 5-class solution included two healthy groups and three unhealthy risk groups. The following results are comparisons between unhealthy and healthy groups.<br>**Sexual minority-specific variables**<br>The "disordered eating + hazardous alcohol use" and the "disordered eating + high exercise" groups showed more discrimination.<br>The "obese + binge eating", "disordered eating + high exercise" groups reported more acceptance concerns.<br>The "obese + binge eating" and "disordered eating + hazardous alcohol use" groups reported a more difficult identity process.<br>The "disordered eating + hazardous alcohol use" groups reported greater concealment and more internalized homonegativity.<br>The "disordered eating + high exercise" and "obese + binge eating" groups reported greater uncertainty.<br>**General variables**<br>The "disordered eating + hazardous alcohol use" and "disordered eating + high exercise" groups reported more negative affect than the healthy classes and the "obese + binge eating" group.<br>The "low health risk + high exercise" group reported more positive affect.<br>The "obese + binge eating", "disordered eating + hazardous alcohol use" and "disordered eating + high exercise" groups reported more social anxiety.<br>The "low health risk + high exercise" group reported more social support from friends.<br>The "disordered eating + hazardous alcohol use" reported less social support from friends than the "low health risk + moderate exercise" and "obese + binge eating" groups.	No significant differences in social support from family among the classes were found.<br>Aside from the "low health risk + high exercise" group, other groups showed no difference in positive affect.
Ancheta et al., 2021	607 cisgender sexual minority women (self-identified as either lesbian, 74,6% or bisexual, 25,4%)	Path analysis	**main variables**: minority stress (stigma consciousness, internalized	Stigma Consciousness Questionnaire modified to add an item for bisexual erasure<br>Internalized Homophobia	**Prevalence**<br>Overeating in the previous 3 months was reported by 17.5% of participants. 9.2% reported an episode of binge eating in the same timeframe.<br>**Path analysis**<br>**Covariates:**	**Path analysis**<br>Direct, indirect, and total associations of internalized homophobia and sexual orientation-based discrimination were nonsignificant.

	18 to 82 years old (mean=39.7)		homophobia, sexual orientation-based discrimination), negative affect (depression), overeating, binge eating; **covariates**: childhood trauma (sexual/physical abuse, parental neglect), body dissatisfaction, sexual identity, BMI	scale adapted from other studies<br>Experiences of Discrimination<br>Ad-hoc questions for categorizing past year depression (at least one depressive episode within one year of the interview date) adapted from the National Institute of Mental Health Diagnostic Interview Schedule<br>Ad-hoc single-item questions for measuring overeating and binge eating adapted from the Diagnostic and Statistical Manual of Mental Disorders IV<br>Eating Disorders Inventory-3 - body dissatisfaction subscale<br>Ad-hoc single-item dichotomous questions for childhood trauma, BMI	Higher body dissatisfaction and higher childhood trauma were associated with higher odds of overeating.<br>**Main variables:**<br>Direct and total effects of stigma consciousness were associated with higher odds of overeating. This association was stronger among Latina women than White and African American women.	There were no significant differences for sexual identity in the direct or indirect associations of minority stress with overeating or binge eating.<br>There were no significant differences in the associations of minority stress with overeating and binge eating between African American and White women.
Convertino et al., 2021	962 sexual minority people (483 women, 479 men, 34.9% gay/lesbian, 58.6% bisexual, 2.1% asexual, 4.4% other) aged 18-30 years (mean=23.7) from the United States	Bivariate correlations, logistic regression model	eating disorders, body dysmorphia, appearance- and performance- enhancing drugs use, internalized homophobia, sexual orientation concealment, heterosexist discrimination, community involvement	Eating Disorder Examination Questionnaire 6.0<br>Dysmorphic Concerns Questionnaire<br>Three items for drug misuse adapted from the Growing Up Today Study<br>Internalized Homophobia Scale Revised<br>Sexual Orientation Concealment Scale<br>Heterosexist Harassment, Rejection, and Discrimination Scale<br>Six items for behavioral community involvement adapted from the Social Justice Sexuality Project	**Bivariate correlations**<br>Internalized homophobia, sexual orientation concealment, heterosexist discrimination, community involvement, drug misuse, positively screening for an ED and for BDD were all positively related.<br>Age was negatively associated with a positive BDD screen and sexual identity.<br>There were negative associations between race and heterosexist discrimination, as well as positive body dysmorphic screen; ethnicity was positively associated with internalized homophobia, heterosexist discrimination, community involvement, positive ED as well as BDD screen and drug misuse.<br>**Logistic regression model**<br>Higher internalized homophobia, sexual orientation concealment, heterosexist discrimination and community involvement were all positively associated with higher odds of eating pathology, dysmorphic concerns, and drug misuse.	**Bivariate correlations**<br>Save for age, sexual identity had no significant relations with any of the other variables.<br>**Logistic regression model**<br>Considering eating pathology, dysmorphic concerns, and drug misuse, there were no significant interactions between internalized homophobia, sexual orientation concealment, heterosexist discrimination and community involvement.
Convertino et al., 2021	962 sexual minority people (483 women, 479 men, 34.9% gay/lesbian, 58.6% bisexual, 2.1% asexual, 4.4% other) aged 18-30 years	Structural Equation Modeling	thin-ideal internalization, muscular-ideal internalization, thinness-based dissatisfaction, muscularity-	Sociocultural Attitudes Towards Appearance Questionnaire 4-Revised<br>Eating Disorder Examination Questionnaire 6.0 gender invariant version<br>Drive for Muscularity	**Structural Equation Model (Men)**<br>The structural model provided acceptable fit to the data and explained 58.4% in muscle-building behaviors and 61.7% of the variance in dietary restraint.<br>The path from the interaction of community involvement and heterosexist discrimination to internalized homophobia was significant. The effect of heterosexist discrimination on internalized homophobia was highest among men with community involvement 1 standard deviation (SD) above the mean.	**Structural Equation Model (Men)**<br>The association between heterosexist discrimination was no longer significantly associated with internalized homophobia among men with community

	(mean=23.7) from the United States		based dissatisfaction, heterosexist discrimination, sexual orientation concealment, internalized homophobia, dietary restraint, muscle building behavior	Scale<br>Internalized Homophobia Scale-Revised<br>Heterosexist Harassment, Rejection, and Discrimination Scale<br>Sexual Orientation Concealment Scale<br>6 items for community involvement adapted from the Social Justice Sexuality Project	**Structural Equation Model (Women)**<br>The structural model for sexual minority women yielded an acceptable fit and explained 75.6% of the variance in muscle-building behaviors and 48.9% of the variance in dietary restraint.<br>Several paths emerged as significant among interaction effects:<br>Interaction between community involvement and pressures to muscular-ideal internalization.<br>Interaction of community involvement and heterosexist discrimination to internalized homophobia.<br>Interaction of community involvement and heterosexist discrimination to sexual orientation concealment.<br>Interaction of community involvement and muscularity-based dissatisfaction to muscularity behaviors.<br>The effect of muscular-ideal internalization was the highest among women with community involvement 1 SD above the mean, and it was not as high in women 1 SD below the mean.<br>The effect of heterosexist discrimination on sexual orientation concealment was higher in women with community involvement 1 SD above the mean, and it was not as high 1 SD below the mean.<br>In women with community involvement 1 SD above the mean, the effect of muscularity-based dissatisfaction on muscularity behaviors was strongest, and the same effect was not as high 1 SD below the mean.	involvement 1 SD below the mean.<br>All other paths were nonsignificant.<br>**Structural Equation Model (Women)**<br>All other paths were nonsignificant.
Cusack et al., 2021	242 transgender and nonbinary people (42.98% trans women, 18.60% nonbinary, 17.77% trans men, 6.61% genderqueer/genderfluid, 4.55% agender, 4.13% women with a trans history, 2.07% men with a trans history, 0.83% bigender; aged 18-70 years old (mean=24.92).	Path analysis	general mental health, minority stress, gender-related rumination, eating-specific rumination, eating disorder psychopathology	Mental Health Screening Test<br>Gender Minority Stress and Resilience Scale<br>Gender Identity Reflection and Rumination Scale<br>Ruminative Response Scale for Eating Disorders<br>Eating Disorder Examination Questionnaire 6	**Descriptive analyses**<br>ED psychopathology did not differ between persons of color and white individuals, or across gender (trans men, trans women, nonbinary)<br>**Bivariate correlations**<br>All main variables were associated at the bivariate level.<br>**Path analysis**<br>A direct effect of gender minority stress was found, with the overall model explaining 62.33% of variance in ED psychopathology.<br>Gender minority stress was associated with gender-related rumination, and ED-specific rumination, with mental health as a covariate.<br>Partial mediation of ED-specific rumination between minority stress and eating disorder psychopathology was found, controlling for BMI and mental health as covariates.	Gender-related rumination was not related to ED psychopathology.
Jhe et al., 2021	498 adults aged 18 to 64 years (mean = 28.50). Sexual orientations were bisexual (74,8%), queer (16,5%), pansexual (3,4%), or other (5,2%, signaling attraction to multiple genders).	Regression analyses	BMI, heterosexist minority stress, anti-bisexual minority stress, emotional eating, body esteem, identity centrality and affirmation, connectedness to the bisexual community	Motivations to Eat Scale - Coping subscale<br>Anti-Bisexual Experiences Scale<br>Heterosexist Harassment, Rejection, and Discrimination Scale - Harassment and Rejection subscales<br>State Self-Esteem Scale - Appearance Self-Esteem subscale<br>Lesbian, Gay, and Bisexual Identity Scale - Identity	**Regression Analyses:** While accounting for sociodemographic variables, BMI, and heterosexist minority stress, anti-bisexual minority stress across all models was associated with more emotional eating and lower body esteem.	**Regression analyses**<br>Analyses testing the two-way moderating interactions were not significant.

				Centrality and Identity Affirmation subscales<br>Ad-hoc item for connectedness to bisexual community		
Panza et al., 2021	55 sexual minority AFAB women (self-identified; 62% bisexual, 33% lesbian, 5% queer; 96% cisgender, 4% genderqueer/gender-nonconforming) aged 18-60 (mean=25) with BMI > 25kg/m². Participants were not enrolled if they were pregnant, had a current serious medical condition, or had a history of weight loss surgery, factors likely to impact hunger and satiety	Generalized linear models	BMI, disordered eating behaviors/attitudes, lifetime experiences of heterosexism, internalized homophobia, sexual orientation concealment, lifetime experiences of sexism, internalized sexism, lifetime experiences of weight stigma, internalized weight bias, overeating, binge eating	Eating Disorder Examination-Questionnaire<br>Heterosexist Harassment, Rejection and Discrimination Scale<br>Lesbian Internalized Homophobia Scale<br>Sexual Orientation Concealment Scale<br>Schedule of Sexist Events<br>Internalized Misogyny Scale<br>Stigmatizing Situations Inventory<br>Weight Bias Internalization Scale<br>Ad-hoc questions for overeating and binge eating	**Sexual orientation minority stress**<br>Women reporting greater baseline sexual orientation concealment endorsed a greater number of Ecological Momentary Assessment (EMA)-measured overeating episodes.<br>Women with greater internalized homophobia reported a greater number of binge episodes during EMA.<br>**Gender-based minority stress**<br>Women endorsing more baseline internalized sexism reported fewer overeating episodes during EMA.<br>**Weight-based minority stress**<br>Women endorsing more lifetime weight stigma events reported more overeating episodes and binge episodes during the EMA period.<br>Women with higher levels of internalized weight bias reported more overeating episodes and binge episodes during EMA	**Sexual orientation minority stress**<br>Heterosexist experiences were not predictive of dysregulated eating during EMA<br>**Gender-based minority stress**<br>Internalized sexism was not predictive of future binge eating and prior sexist events were not predictive of any future dysregulated eating behaviors.
Uniacke et al., 2021	287 transgender and gender nonbinary people	Hierarchical multivariable logistic regression	transgender congruence, receipt of gender-affirming care, minority stress, disordered eating symptoms	Eating Disorder Examination - weight concern and restraint subscales<br>Transgender Congruence Scale<br>Everyday Discrimination Scale<br>Stigma Consciousness Questionnaire<br>Transgender Identity Survey<br>Brief Symptom Inventory - anxiety subscale	**Prevalence**<br>53.3% of participants reported a lifetime history of more than one disordered eating behavior. The most prevalent lifetime and current behavior reported was Loss-Of-Control (LOC) eating (34.1% of individuals reported more than one episode during the previous month, 14.9% reported more than four episodes). 3.8% of participants reported use of laxatives, diuretics, or other medications to control shape or weight during the previous month, and 2.8% reported the same on more than four occasions. 3.5% of participants reported self-induced vomiting in the previous month and 1.7% had induced vomiting more than four times. Finally, 7.3% of participants reported compulsive exercise during the previous month and 3.5% endorsed more than four episodes of the same. 56.4% of participants reported overvaluation of weight in the previous year, and 18.8% reported beliefs that they were fat despite other people reporting they were too thin. 60.6% of participants reported shape and weight concerns that were linked to eating behavior and reported attempts to reduce their intake to affect their shape or weight during the previous year.<br>**Predictors of eating-related psychopathology**<br>Three statistical models were analyzed.<br>In Model A, anxiety was associated with a higher chance of eating-related psychopathology. In Model B, higher transgender congruence was associated with a lower chance of eating-related psychopathology – participants who reported more congruence between their gender identity and external appearance had a lower chance to report eating-related symptoms. Additionally, individuals who reported receiving gender-affirming psychotherapy during the previous year had a higher chance to report eating-related symptoms. In Model C, gender-affirming psychotherapy in the	**Prevalence**<br>**Differences between groups:**<br>The groups did not otherwise differ in the presence or frequency of disordered eating symptoms.<br>**Predictors of eating-related psychopathology**<br>In model B, neither prior gender-affirming surgery nor hormone therapy during the past year were associated with current eating-related psychopathology.<br>**Differences between groups:**<br>Bivariate comparison of clinical characteristics, including age, BMI, sex assigned at birth, race/ethnicity, receipt of past-year gender-affirming medical or psychotherapeutic care, and factors related to identity development and minority stress, did not significantly differ between individuals with and without persistent symptoms at follow-up.

					previous year and increased internalized transphobia were associated with a higher chance of reporting eating-related psychopathology.	
Barnhart et al., 2022	1051 self-identified sexual minority people. 519 men (469 gay men and 50 bisexual men, 532 women (317 lesbian women and 215 bisexual women). Aged 18 to 49 years old.	Structural Equation Modeling	sexual orientation, appearance pressures, thinness-ideal internalization, muscularity-ideal internalization, drive for muscularity, body image comparisons, disordered eating, internalized homophobia, sexual orientation concealment, bmi, age	Sociocultural Attitudes Towards Appearance Questionnaire 4-Revised<br>Drive for Muscularity Scale<br>Female Muscularity Scale<br>Upward Physical Appearance Comparison Scale and Downward Appearance Comparison Scale<br>Eating Disorder Examination Questionnaire-Short<br>Revised Internalized Homophobia Scale<br>Self-Concealment Scale	**Structural Equation Modeling**<br>**Sexual minority men:**<br>A structural model and a revision of the same model were analyzed. The revised one showed an acceptable fit.<br>The revised model explained 80.4% of the variance in drive for muscularity and 58.9% of the variance in thinness-oriented disordered eating.<br>The largest direct effects on following variables were presented by appearance pressures.<br>Downward body image comparison was associated with thinness-oriented disordered eating and drive for muscularity.<br>Sexual orientation concealment was negatively associated with thinness-oriented disordered eating and drive for muscularity, suggesting the existence of suppression effects. Internalized homophobia was positively associated with thinness oriented disordered eating.<br>**Sexual minority women:**<br>Acceptable model fit. The model explained 54.0% of the variance in thinness-oriented disordered eating and 73.1% of the variance in drive for muscularity.<br>The largest direct effects on following variables were presented by appearance pressures.<br>Thinness-ideal internalization was associated with upward body image comparisons.<br>Downward body image comparisons were associated with thinness oriented disordered eating.<br>Upward body image comparisons were associated with drive for muscularity.	**Sexual minority men:**<br>Upwards body comparison were not related to drive for muscularity and thinness-oriented disordered eating.<br>**Sexual minority women:**<br>Thinness-ideal internalization was not associated with downward body image comparisons.<br>Muscularity-ideal internalization was not associated with upward or downward body image comparisons.<br>Upwards body image comparison were not related to thinness-oriented disordered eating.<br>Downward body image comparisons were not associated with drive for muscularity.<br>Sexual orientation concealment and internalized homophobia were not significantly associated with thinness-oriented disordered eating or drive for muscularity.
Brokjøb et al., 2022	85 norwegian transgender and nonbinary participants (53 men, 18 women, 14 nonbinary) between 18 and 59 years old	Mediation analysis	minority stress, gender dysphoria, anxiety, depression, eating pathology	Daily Heterosexist Experiences Questionnaire<br>Gender Preoccupation and Stability Questionnaire<br>Eating Disorder Examination Questionnaire Short<br>Patient Health Questionnaire-9<br>General Anxiety Disorder-7 Questionnaire	In mediation analyses, an age-group split was employed to separate results for younger (18-22) and older (23-59) people.<br>**Younger group**<br>Minority stress accounted for 22% of the variation in eating pathology scores.<br>Mediation analyses found that minority stress predicted all psychopathologies (anxiety, depression, eating pathology) directly when accounting for gender dysphoria.<br>**Older group**<br>In the older group, gender dysphoria significantly contributed to explaining the variation in eating pathology scores and accounted for 24% of the variation in eating pathology scores.<br>Minority stress predicted gender dysphoria, and, accounting for minority stress in the model, gender dysphoria predicted rates of all psychopathologies (anxiety, depression, eating pathology). A significant indirect effect was found for all psychopathologies. When mediating for gender dysphoria there was no evidence that minority stress independently predicted any of the examined psychopathologies. This suggests a full mediation of gender dysphoria in the relationship between minority stress and all examined psychopathologies.	No significant differences in psychopathology rate were found between the younger and older groups in any of the scores in the sample (anxiety, depression, eating pathology). There were no differences in the minority stress and gender dysphoria scores either.<br>**Younger group**<br>Introducing gender dysphoria in the regression model for the younger group did not contribute further to explaining the variation in eating pathology scores and was not a significant predictor.<br>There were no indirect effects of minority stress on any of the examined psychopathologies. This suggests no mediating effects of gender dysphoria in the relationship between minority stress and any of the examined psychopathologies.

						**Older group**<br>Minority stress did not contribute to the regression model for the older group.
Muratore et al., 2022	93 self-identified Transgender and Gender-Expansive adults over 18 years old (43 women, 31 men, 7 nonbinary)	Multiple Regression analyses	tripartite influence model factors (family, media, appearance influences), gender minority stressors, appearance comparison, body ideal internalization, discrimination, victimization, rejection, non-affirmation, internalized transphobia, negative expectations, concealment, community connectedness	Gender Minority Stress and Resilience Measure<br>Sociocultural Attitudes Towards Appearance Questionnaire–4<br>Perceived Sociocultural Pressures Scale<br>Physical Appearance Comparison Scale<br>Body-Image Ideals Questionnaire<br>Eating Attitudes Test<br>Drive for Muscularity Scale	**Correlation coefficients**<br>Minority stress factors were moderately, positively correlated with the Tripartite Influence Model factors.<br>Minority stress factors were moderately, positively correlated with body dissatisfaction and disordered eating.<br>Community connectedness was moderately, negatively correlated with some Tripartite Influence Model factors and disordered eating.<br>Pride was moderately connected with Tripartite Influence Model factors and disordered eating.<br>Tripartite Influence Model factors were strongly positively correlated with disordered eating.<br>**Test of Hypothesis 1**<br>Minority stress factors accounted for 33% of the variance in body dissatisfaction, 59% in thinness-oriented and 42% of muscularity-oriented disordered eating.<br>**Test of Hypothesis 2**<br>Muscularity-oriented factors explained 21% of the variance in body dissatisfaction and 65% of the observed variance in muscularity-oriented disordered eating<br>Thinness-oriented factors explained 58% of the observed variance in disordered eating.<br>**Test of Hypothesis 3**<br>Adding gender minority stress factors to tripartite influence factors explained significant unique variance in body dissatisfaction in the thinness-oriented and muscularity-oriented models. Thinness-oriented models explained 38% of body dissatisfaction and 70% of thinness-oriented eating. Muscularity-oriented models explained 47% of body dissatisfaction and muscularity-oriented disordered eating.	**Correlation coefficients**<br>No significant association was found between body dissatisfaction and disordered eating.<br>Age was not associated with any study outcome.<br>**Test of Hypothesis 2**<br>Overall relationship between thinness-oriented factors and body dissatisfaction was not significant<br>**Test of Hypothesis 3**<br>Adding gender minority stress factors to tripartite influence factors did not explain the significant amounts of added variance in two disordered eating models and two body dissatisfaction models.
Roberts et al., 2022	8814 LGBTQ English-speaking self-identified sexual and gender minority adolescents (aged 13-17) from the United States	Chi squared tests, logistic regression models, simple slope analyses	sexual orientation, gender identity, disordered eating, self-esteem, stress, depression, feelings about one's SGM identity, stress of coming out	ad-hoc questions for disordered eating surveying five behaviors: caloric restriction, diet pills, taking laxatives, objective binge eating<br>ad-hoc question for average level of stress<br>Rosenberg Self-Esteem Scale<br>Kutcher's Adolescent Depression Scale<br>ad-hoc questions for feelings about one's SGM identity, surveying positive feelings about SGM identity, access to SGM resources, future beliefs about life as an SGM person, openness about SGM	**Prevalence**<br>16,5% of adolescents met the clinical threshold for at least one disordered eating behavior. Most common clinical-threshold behavior was binge eating (9,73%).<br>**Associations**<br>Self-esteem was associated with lower odds of clinical threshold caloric restriction, purging and binge eating.<br>Depressive symptoms were associated with higher odds of clinical threshold caloric restriction, diet pill use, purging, laxative use and binge eating.<br>Positive feelings about being SGM were associated with lower odds of clinical threshold caloric restriction, purging and binge eating.<br>Openness with one's SGM identity was associated with lower odds of clinical threshold caloric restriction.<br>In adolescents who were out, higher stress of coming out was associated with higher odds of clinical threshold caloric restriction, purging and binge eating.<br>**Moderation models**<br>Self-esteem was associated with lower odds of clinical threshold caloric restriction for both SM and GM adolescents.<br>Depression was associated with higher odds of clinical threshold caloric restriction for both SM and GM adolescents.<br>General stress was associated with higher odds of clinical threshold caloric	**Associations**<br>General stress' associations with disordered eating behaviors were nonsignificant<br>**Moderation models**<br>General stress was not associated<br>**SGM-specific factors**<br>Differences in the associations between SGM-specific factors and disordered eating did not emerge for SM versus GM youth.<br>Access to SGM resources was not associated with disordered eating.

				identity<br>ad-hoc questions for stress of coming out, surveying the levels of perceived stress in 10 different coming out experiences	restriction among GM adolescents only.<br>**SGM-specific factors**<br>More positive feelings about SGM identity were associated with lower odds of clinical threshold caloric restriction, purging, and binge eating among both SM and GM adolescents. Further, openness about one's SGM identity was associated with lower odds of clinical threshold caloric restriction for both SM and GM youth.<br>For SGM adolescents who had "come out" to others, higher stress of coming out was associated with higher odds of clinical threshold caloric restriction, purging, and binge eating.	
Schmidt et al., 2022	216 men (112 gay men and 104 heterosexual men)	Chi-squared tests, t-tests, Spearman's correlation coefficient	body image, disordered eating, discrimination, identification and involvement with the gay community	Body Appreciation Scale-2<br>Bodybuilder Image Grid-Original<br>Body Image Coping Strategies Inventory<br>Body Image Disturbance Questionnaire<br>Dysmorphic Concern Questionnaire<br>Drive for Leanness Scale<br>Drive for Muscularity Scale<br>Drive for Thinness Scale<br>Eating Disorder Examination-Questionnaire<br>Gender-Neutral Body Checking Questionnaire<br>Identification and Involvement with the Gay Community Scale<br>Everyday Discrimination Scale	**Prevalence**<br>Gay men showed significantly higher scores in terms of drive for thinness, appearance fixing and general body image disturbance compared to heterosexual men.<br>Gay men scored significantly higher than heterosexual men in terms of total eating disorder pathology, as well as weight, shape and dysmorphic concerns scales.<br>**Correlations**<br>Everyday discrimination of gay men was positively correlated with body avoidance, general body image disturbance, total eating disorder pathology, weight, shape and dysmorphic concerns.	**Prevalence**<br>There were no significant differences between gay men and heterosexual men in terms of drive for leanness, body avoidance and body checking.<br>There were no differences between gay men and heterosexual men regarding restraint eating and eating concern.<br>There was no difference between gay men and heterosexual men regarding the frequency of everyday discrimination experiences.<br>**Correlations**<br>There was no relationship between involvement with the gay community and body image, eating disorder or body dysmorphic disorder factors.<br>All other relationships were non-significant.
Serier et al., 2022	1363 post-9/11 U.S. veterans; 805 women (656 heterosexual, 51 lesbian, 98 bisexual) and 558 men (540 heterosexual, 11 gay, 7 bisexual); self-identified trans, genderqueer or other gender identity categories excluded due to sample size too small to analyze	Correlational analyses, logistic regression models	disordered eating, combat trauma exposure, discrimination	Eating Disorder Diagnostic Scale<br>Clinical Impairment Assessment<br>Deployment Risk and Resilience Inventory<br>Everyday Discrimination Scale	**Prevalence**<br>10% of lesbian women met criteria for probable eating disorder diagnosis; 12% scored above the clinical cut-off.<br>23,4% of bisexual women met criteria for probable eating disorder diagnosis, 42,8% scored above the clinical cut-off.<br>Approximately 38% of lesbian and 14% of bisexual women reported discrimination based on sexual orientation in the military.<br>Approximately 27% of gay and 29% of bisexual men reported discrimination based on sexual orientation.<br>**Regression analyses**<br>In women, identifying as bisexual was associated with higher scores on the eating disorder and clinical impairment scales.<br>Lesbian women reported DE symptoms comparable to heterosexual women.<br>Having experienced any sexual trauma was associated with elevated scores on eating disorder and clinical impairment scales.<br>Identifying as bisexual was associated with greater odds of endorsing regular self-induced vomiting and fasting.<br>**Correlational analyses**	**Prevalence**<br>Samples for gay and bisexual men were too small for non-exploratory analysis<br>**Regression analyses**<br>Combat trauma was not associated with elevated scores on eating disorder and clinical impairment scales.<br>When including trauma exposure into the model, the association between bisexual identity and endorsing regular fasting became non-significant.

					In heterosexual and bisexual women and heterosexual men, discrimination in the military was positively associated with eating disorder scores.	
Simone et al., 2023	105 queer women (40,4% lesbian, 25% bisexual, 10,5% pansexual, 15,4% queer, 3,8% asexual; mean age 23,6 years)	Reflexive thematic analysis	race, gender identity, sexual orientation, disordered eating, weight perception, weight control, body image ideal	Eating disorder Examination-Questionnaire<br>Open-ended questions regarding weight perception, weight control and body image ideals	**Prevalence**<br>69,5% of participants scored above the clinical cut-off score for global eating disorder symptomatology, 57,6% scored above the cut-off for eating restraint, 57% on eating concern, 81% on shape concern.<br>44,2% of participants reported that gender identity influenced their weight concerns; 39,4% reported the same for weight control behaviours. 39,4% of participants reported that their sexual orientation influenced their weight concern, 37,5% reported the same for weight control behaviors, 32,7% reported the same for body image ideals.<br><br>**Thematic analysis**<br>Analysis of the open-ended questions resulted in 9 emerging themes.<br>Among these, several recounted the influence of sexual orientation and gender identity on body image and disordered eating.<br>One emerging theme highlighted participants' experiences of trying to move towards idealized body images to compensate for aspects of identity (queerness, weight) linked to systems of oppression. One participant in particular recounts fear of rejection due to queerness status.<br>Another theme is the entwinement of gender identity with weight status, connecting femininity and low body weight.<br>Another is the use of weight as 'queer signaling', describing participants' need to attain specific and restrictive body shapes.<br>Queer community is described as body positive.<br>Queer-specific body ideals and norms are described as restrictive depending on specific sub-cultural ideals (e.g. masculine, feminine, androgynous ideals; butch, dyke, small ideals)	N/A
Urban et al., 2023	212 transgender and nonbinary people over 18 years old who were experiencing disordered eating or had an eating disorder (146 nonbinary people, 80 trans men, 28 with a self-identified gender, 14 trans women; 59% queer, 22,6% bisexual, 13,7% gay, 10,8% lesbian; mean age 27 years)	Linear multiple regressions	gender dysphoria, discrimination trauma, internalized transphobia, eating pathology	Trauma Symptoms of Discrimination Scale<br>Transgender Identity Scale<br>Utrecht Gender Dysphoria Scale - Gender Spectrum<br>Eating Disorder Examination Questionnaire<br>Insufficient Effort Responding Detection Scale	**Bivariate Correlations**<br>Moderate to weak positive correlations were found between all variables and eating pathology.<br><br>**Multiple regression models**<br>In the first model, discrimination trauma and internalized transphobia were significant and positive predictors of eating pathology. The model predicted 15,4% of variance in eating pathology.<br>In the second model, internalized transphobia mediated the path between discrimination trauma and eating pathology. Its effect explained 10,3% of discrimination trauma on eating pathology.<br>In the third model, the effect of discrimination trauma on internalized transphobia was moderated by levels of gender dysphoria.	**Multiple regression models**<br>In the first model, gender dysphoria was not a significant predictor of eating pathology.
Hong et al., 2023	200 transgender people aged 18-39 (30% men, 57,5% women, 6% genderqueer, 1,5% gender non-conforming, 1%	bivariate correlations, regression analyses, path analysis	transgender identity, internalized cisgenderism, body surveillance, body shame, psychological	Revised Internalized Homophobia modified for transgender populations<br>Objectified Body Consciousness Scale<br>Short Kessler Psychological Distress Scale	**Bivariate correlations**<br>Age was negatively associated with body surveillance, body shame, suicidal ideation, and suicide attempts (all effect sizes small). BMI was associated with disordered eating (small effect size).<br>Internalized cisgenderism yielded associations with body shame (medium effect), psychological distress (medium effect), disordered eating (medium effect), NSSI (small effect), suicidal ideation (small effect), and suicide attempts (small effect).	**Path analysis**<br>Path from internalized cisgenderism to body surveillance.

	gender-fluid, 3,5% non-binary)		distress, disordered eating, non-suicidal self injury, suicidal ideation and suicide attempts	Eating Disorder Examination Questionnaire - Short form<br>Non-suicidal self-injury scale	Body surveillance had associations with body shame (medium effect), psychological distress (medium effect), disordered eating (small effect), and suicidal ideation (small effect). Body shame was associated with psychological distress (medium effect), disordered eating (large effect), NSSI (small effect), suicidal ideation (small effect), and suicide attempts (small effect).<br><br>**Regression analyses**<br>Internalized cisgenderism was associated with higher body shame, psychological distress, disordered eating, NSSI, and suicidal ideation. Higher body surveillance was associated with body shame and psychological distress; higher body shame was associated with psychological distress, disordered eating, NSSI, and more suicide attempts.<br><br>**Path analysis**<br>Body shame had significant indirect effects with body surveillance, disordered eating, NSSI, suicide attempts.<br>Body surveillance, in turn, was associated with psychological distress, disordered eating, NSSI and suicide attempts.<br>Internalized cisgenderism was associated with higher body shame, which, in turn, was associated with psychological distress, disordered eating, NSSI and suicide attempts.	
